# Urine dipstick for screening plasma glucose and bilirubin in low resource settings: a proof-of-concept study

**DOI:** 10.1515/almed-2023-0114

**Published:** 2023-10-20

**Authors:** Laura Pighi, Davide Negrini, Brandon M. Henry, Gian Luca Salvagno, Giuseppe Lippi

**Affiliations:** Section of Clinical Biochemistry and School of Medicine, University of Verona, Verona, Italy; Clinical Laboratory, Division of Nephrology and Hypertension, Cincinnati Children’s Hospital Medical Center, Cincinnati, OH, USA

**Keywords:** dipstick, plasma, test strip, urine, plasma

## Abstract

**Objectives:**

The purpose of this proof-of-concept study was to investigate whether a commercially available urine dipstick may provide potentially useful information for screening plasma glucose and bilirubin in human plasma samples.

**Methods:**

Glucose and bilirubin were assayed in 60 anonymized lithium-heparin residual plasma samples using the Roche COBAS 8000 or after pipetting 10 µL of plasma onto the pads of a commercial urine dipstick. Semiquantitative urine test results obtained with the dipstick were directly compared to paired test results obtained with COBAS.

**Results:**

Median plasma glucose values between COBAS and dipstick were slightly different (5.8 vs. 5.6 mmol/L; p=0.040), while no significant difference was found in bilirubin values between COBAS and dipstick (11.2 vs. 8.6 μmol/L; p=0.090). The Spearman’s correlation between COBAS and dipstick was 0.83 (95% CI, 0.73–0.90; p<0.001) for plasma glucose and 0.78 (95% CI, 0.66–0.87; p<0.001) for plasma bilirubin, respectively. Cumulative agreement between COBAS and dipstick was high for both glucose (88%; kappa statistic statistics, 0.75; 95% CI, 0.58–0.92; p<0.001) and bilirubin (88%; kappa statistics, 0.76; 95% CI, 0.60–0.92; p<0.001).

**Conclusions:**

The results of this proof-of-concept study indicate that the commercial urine test strip used in our study provides acceptable performance for screening plasma glucose and bilirubin levels compared with reference laboratory assays.

## Introduction

A urine dipstick is a simple diagnostic tool, commonly used in medicine, that can be used to quickly assess a number of urine components. It is essentially based on a thin plastic strip containing many different squares or pads, each containing a specific reagent [1]. When the strip is dipped into a urine specimen, the pads can change their basic colors, indicating the presence or even the (semi-quantitative) concentration of certain substances in the urine. Modern dipsticks can either be read optically, i.e. by visually comparing the color changes on the individual pads with a reference scale or table usually supplied by the manufacturer in the dipstick box, or they can be read automatically by modern urinalysis equipment [2]. Importantly, while the test strips do not provide a definitive diagnosis, they can be considered a rapid and convenient approach to patient screening, particularly in very low-resource [3] or difficult-to-access (i.e., for catastrophes or natural disasters) [4] settings, where laboratory instrumentation and/or point-of-care devices may also be unavailable.

Although the use of dipsticks has traditionally been reserved for examination of urine samples, there is a tantalising hypothesis that they could also be used for screening serum or plasma, making it possible to obtain, rapidly and relatively inexpensively, semi-quantitative information on the concentration of some important analytes in these biological matrices such as glucose, total proteins, urobilinogen, bilirubin, creatinine, pH, blood, ketones, nitrites, leukocytes, and specific gravity. Analytes whose concentrations may overlap between urine and plasma, and which could hence be more diagnostically useful in screening patients include, in particular, glucose and bilirubin. To this end, this proof-of-concept study was designed to investigate whether a commercial urine dipstick may provide some potentially useful information for screening plasma glucose and bilirubin.

## Materials and methods

We randomly selected 60 anonymized lithium-heparin residual samples (3.5-mL lithium heparin blood tube; Vacutest Kima, Padova, Italy) from the morning of one routine working day in the Service of Laboratory Medicine of the University Hospital of Verona, after a clinical chemistry profile (including glucose, protein, bilirubin, and creatinine) was conducted on a Roche Cobas 8000 (Roche Diagnostics, Basel, Switzerland). The identity of the specimen was replaced with new identifiers to prohibit identifiability of the data set, but remaining suitable for processing and data analysis. Immediately after sample release by the analyzer, 10 µL of plasma were applied with a micropipette to each of the 10 pads of a commercial urine dipstick (AUTION Sticks, Arkray, Kyoto, Japan), the characteristics of which have been comprehensively reported elsewhere [5]. After 10 s, the excess plasma was carefully removed by placing the strip on a piece of tissue paper, and the test results were visually interpreted within 60 s by comparing the color of the pad with the color indicated in the chart on the bottle. Two experienced laboratory personnel interpreted all visual readings, and any discrepancies were clarified by a third person. The following parameters were evaluated on the test strip: glucose (glucose oxidase-peroxidase chromogen reaction; range of measurement: 50–1,000 mg/dL, i.e., 2.8–55.6 mmol/L), and bilirubin (diazonium salt colorimetric reaction; range of measurement:0.5–6.0 mg/dL; 8.55->102.6 μmol/L). Unfortunately, we were unable to test even total proteins (protein-error reaction of pH indicator, range of measurement: 10–1,000 mg/dL; 0.1–10) and creatinine (Jaffe reaction, range of measurement:10–300 mg/dL; i.e., 884.2–26.526) due to their too low and too high measurement ranges in the dipstick, respectively.

All test results were expressed as median and interquartile range (IQR). Semi-quantitative urine test results obtained on the urine dipstick were directly correlated with paired sample test results on the Roche Cobas 8000 by Mann–Whitney U tests and Spearman’s correlation. We also estimated the concordance between discrete categories of glucose and bilirubin plasma levels assayed with the two techniques as follows: glucose: 0–4.1, 4.2–8.1, 8.2–13.9, >13.9 mmol/L; bilirubin: 0–17.0; 17.1–51.3; >51.3 μmol/L. The statistical analysis was conducted using Analyse-it (Analyse-it Software Ltd, Leeds, UK). The study was performed on anonymized remnant patient samples after routine testing was completed, such that informed consent was not required. The study was conducted in accordance with the Declaration of Helsinki and in compliance with relevant local legislation and was cleared by the Ethics Committee of the University Hospital of Verona (970CESC; July 20, 2016).

## Results

**Figure 1:**
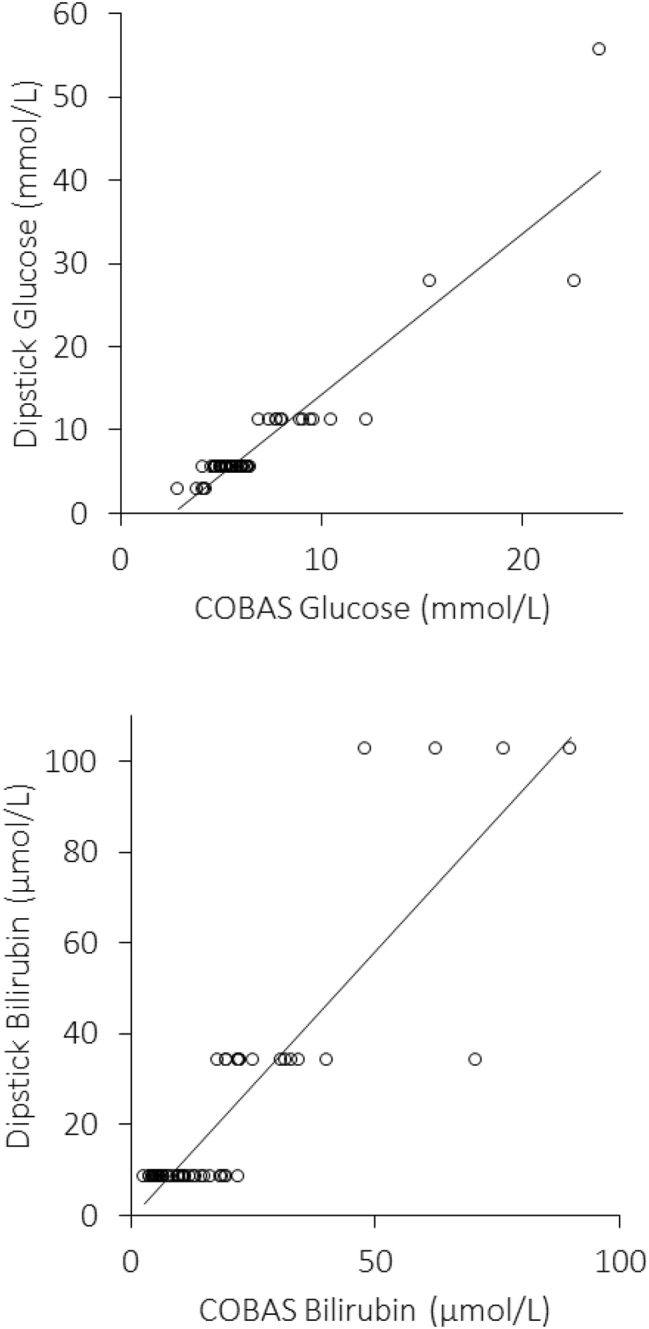
Spearman’s’ correlation of plasma glucose and bilirubin test results between Roche COBAS 8000 and manual reading of commercially available urine dipstick.

The main results of this study are shown in Table 1 and Figure 1. A marginally significant difference was observed in median plasma glucose values between COBAS (5.8 mmol/L; IQR, 5.0–6.6 mmol/L) and dipstick (5.6 mmol/L; IQR, 5.6–6.9 mmol/L; p=0.040), whilst no significant difference could be observed in median plasma bilirubin levels between COBAS (11.2 μmol/L; IQR, 6.6–22.0 μmol/L) and dipstick (8.6 μmol/L; IQR, 8.6–34.2 μmol/L; p=0.090), respectively. The Spearman’s correlation between COBAS and dipstick was 0.83 (95% CI, 0.73–0.90; p<0.001) for plasma glucose and 0.78 (95% CI, 0.66–0.87; p<0.001) for plasma bilirubin, respectively. The cumulative concordance between COBAS and dipstick was high for both glucose (88%; kappa statistics, 0.75; 95% CI, 0.58 to 0.92; p<0.001) and bilirubin (88%; kappa statistics, 0.76; 95% CI, 0.60 to 0.92; p<0.001) (Table 2).

**Table 1:** Comparison of plasma glucose and bilirubin test results between Roche COBAS 8000 and manual reading of commercially available urine dipstick.

Parameter	Roche COBAS 8000	Dipstick	p-Value
Glucose, mmol/L	5.8 (IQR, 5.0–6.6)	5.6 (IQR, 5.6–6.9)	0.040
Bilirubin, µmol/L	11.2 (IQR, 6.6–22.0)	8.6 (IQR, 8.6–34.2)	0.099

**Table 2:** Concordance of glucose and bilirubin plasma values measured with Roche COBAS 8000 and with a commercially available urine dipstick.

Glucose, mmol/L	Dipstick				
COBAS	From 0 to 4.1	From 4.2 to 8.1	From 8.2 to 13.9	>13.9	Total
From 0 to 4.1	4	1	0	0	5
From 4.2 to 8.1	1	39	5	0	45
From 8.2 to 13.8	0	0	7	0	7
>13.9	0	0	0	3	3
Total	5	40	12	3	60

Bilirubin, µmol/L	Dipstick			
COBAS	From 0 to 17.0	From 17.1 to 51.3	>51.3	Total
From 0 to 17.0	37	0	0	37
From 17.1 to 51.2	5	13	1	19
>51.3	0	1	3	4
Total	42	14	4	60

## Discussion

In very resource-poor areas, as well as in areas affected by catastrophic emergencies such as wars, earthquakes, tsunamis, floods, forest fires, etc. [6], access to diagnostic testing can be a major challenge for a variety of reasons, including limited financial resources, inadequate or inaccessible diagnostic tools and/or infrastructures, or lack of dedicated personnel. Although efforts could be made to improve or restore the availability of diagnostic testing in these disrupted settings, the challenges are often overwhelming [6]. The availability of manual, rapid, and inexpensive tests that can provide a (even semi-quantitative) result of some key laboratory tests can therefore be considered an extremely valuable prospect. To this end, we designed this proof-of-concept study to investigate whether conventional dipsticks that can be visually interpreted by health care professionals or by patients themselves can temporarily substitute for the use of more accurate diagnostic tests and thus provide some basic diagnostic data that could initially guide diagnostic considerations and clinical decision making.

Overall, the results of our proof-of-concept study suggest that the commercially available urine dipstick that we used in our study provides acceptable performance in screening plasma glucose and bilirubin levels compared with the reference laboratory assays. We limited our analysis to these two parameters because the ranges of the other parameters included in the test strip do not overlap between plasma and urine. Therefore, in cases where it is extremely necessary or imperative to obtain additional information about a patient’s condition but laboratory analyzers are not available, the urine dipstick could be used provisionally to screen serum or plasma glucose and bilirubin levels if simple means of sample separation are available (e.g., small and relatively inexpensive manual centrifuges, which can be purchased for around 100 euros). These results could also pave the way for the development of specific plasma-, serum-, or even whole-blood-based diagnostic strips that could be used in locations where conventional laboratory testing (either with traditional laboratory analyzers or with point-of-care equipment) cannot be performed temporarily or permanently for a variety of reasons. It is undeniable that there are many point-of-care tests (POCT) that can be used to perform the same tests. Nevertheless, they are not free, and even if the single test has a comparable price, the additional cost of the instrumentation would exceed that of the manual test strip, which could only be interpreted by visual inspection. Our proof-of-concept study is therefore best suited for use in circumstances or environments where laboratory equipment is not readily available and/or cannot be purchased for economical constrains.
